# A Rare Cause of Hemophagocytic Lymphohistiocytosis:* Fusobacterium* Infection—A Case Report and Review of the Literature

**DOI:** 10.1155/2016/4839146

**Published:** 2016-08-03

**Authors:** Ghulam Rehman Mohyuddin, Heather J. Male

**Affiliations:** ^1^Kansas University Medical Center, 3901 Rainbow Boulevard, Kansas City, KS 66160, USA; ^2^Division of Hematologic Malignancies and Cellular Therapeutics (HMCT), Department of Internal Medicine, University of Kansas Cancer Center, 2330 Shawnee Mission Parkway, Mailstop 5003, Westwood, KS 66205, USA

## Abstract

Hemophagocytic lymphohistiocytosis (HLH) is a rare syndrome characterized by excessive activation of the immune system. Bacterial infections are very rare precipitants of this disease. A 19-year-old gentleman presented with headache, fatigue, and malaise. He was found to be hypotensive, tachycardic, and febrile. Broad spectrum antibiotics were initiated, and a lumbar puncture ruled out meningitis. Patient progressively developed shock that required use of vasopressors, as well as renal and respiratory failure. Blood cultures grew* Fusobacterium necrophorum*. Given continued fevers despite appropriate antimicrobials, a bone marrow biopsy was performed revealing increased histiocytes with hemophagocytosis. Dexamethasone was added with dramatic clinical improvement. Our case highlights* Fusobacterium* as a rare precipitant of HLH and proves that a high index of clinical suspicion is crucial for early diagnosis of HLH, allowing for prompt initiation of HLH-specific immunosuppressive therapy that can be life-saving.

## 1. Introduction

Hemophagocytic lymphohistiocytosis (HLH) is a rare syndrome characterized by excessive activation of the immune system. Viral infections are a common trigger, but bacterial infections are rare precipitants [1]. We present a case of HLH associated with* Fusobacterium* infection, which has only been reported once previously [2].

## 2. Case Presentation

A 19-year-old previously healthy male acutely developed headache, photophobia, diffuse myalgia, fatigue, nausea, and vomiting. Upon presentation he was found to be hypotensive, tachycardic, and febrile. He was given intravenous fluids and started on vancomycin and ceftriaxone. A lumbar puncture was not suggestive of meningitis. A Computed Tomography (CT) of the head was negative for any pathology, and CT of the abdomen/pelvis showed multifocal septic pulmonary emboli, enteritis, possible hepatic hemangioma, and splenomegaly. He developed respiratory failure requiring intubation and shock requiring use of vasopressors within a day of arrival. Blood cultures grew* Fusobacterium necrophorum*. His renal function worsened, and he was transferred to our tertiary care institution on hospital day 2.

The patient's initial evaluation was notable for recurrent high fevers, high ventilator support requirements, tachycardia, and reduced cardiac systolic function. Laboratory values were significant for anemia (Hb 10.9 gm/dL), thrombocytopenia (platelet count 65 K/*μ*L), leukocytosis (WBC 13.6 K/*μ*L) with left shift, and elevated D-dimer. Chemistry was significant for creatinine 4.37 mg/dL, BUN 52 mg/dL, total bilirubin 3.1 mg/dL, direct bilirubin 2.3 mg/dL, and uric acid 16.0 mg/dL. Ferritin was 690 ng/mL and triglycerides were 488 mg/dL. Other investigations were unremarkable.

Given continued fevers despite appropriate antimicrobials, a bone marrow biopsy was performed on hospital day 4, which revealed normocellular marrow with increased histiocytes with hemophagocytosis and no evidence of malignancy.

Due to persistent high oxygen requirements, fevers, and renal failure despite therapy, dexamethasone dosed according to the pediatric HLH-94 protocol at 10 mg/m^2^ daily was initiated on day 6.

A significant improvement in inflammatory markers and renal and hepatic function was noted within 24 hours of starting dexamethasone. The patient was quickly weaned from mechanical ventilation. The hepatic lesion suspicious for hemangioma was drained and found to be purulent, although cultures remained negative. Ferritin peaked at 966 ng/mL and triglycerides peaked at 725 mg/dL, both normalizing within 2 weeks. Subsequent evaluation showed resolution of the cardiac dysfunction, septic emboli, and hepatosplenomegaly.

The patient was discharged home with a dexamethasone taper and an extended course of ertapenem.

## 3. Discussion

Diagnosing HLH requires a high index of clinical suspicion, as the presentation is often nonspecific. The criteria for diagnosis are well published, but a clinical diagnosis remains [3].

Although severe infection alone could have explained the multisystem organ failure and hemophagocytosis, our patient did meet 5/8 criteria for HLH. The possibility of HLH was entertained early in our patient's course because the immune response and the multisystem organ failure despite antimicrobial therapy exceeded what would be expected in a healthy 19-year-old.

In the adult population, an elevated ferritin is not specific for HLH, as renal failure, hepatocellular injury, malignancies, and infections can cause a drastically elevated ferritin level [4, 5]. In our case, we feel we diagnosed HLH early enough to prevent progressive macrophage activation, and thus the ferritin only peaked at 966, a value far lower than that seen in most cases [1, 6, 7].

The premise for treatment of HLH is to stop the trigger and control the overactive immune system. Any underlying cause such as infection or malignancy should be aggressively treated, with consideration of additional immunosuppressive therapy only if there is insufficient improvement. Treatment in adults is largely based on the HLH-94 study in which pediatric patients were treated with an 8-week induction of dexamethasone and etoposide [8]. Etoposide based regimens are now considered the standard of care for HLH. The ongoing HLH 2004 trial is evaluating the addition of Cyclosporine to that regimen [9].

In certain cases of infection-associated HLH, glucocorticoid monotherapy has been used with success, with the caveat that there will be a low threshold to proceed to full immunosuppressive therapy if a satisfactory response is not achieved rapidly [10, 11].

## 4. Conclusion

This case report highlights that with early identification of HLH in infection, it can be stopped with steroids alone. It describes the rare association between* Fusobacterium* infection and secondary HLH. We conclude by emphasizing that a high index of clinical suspicion is crucial for early diagnosis of secondary HLH, and prompt initiation of HLH-specific immunosuppressive and anti-inflammatory therapy in addition to treatment directed against the infectious source can be life-saving in infection-associated HLH.
